# Smoothened Agonist Reduces Human Immunodeficiency Virus Type-1-Induced Blood-Brain Barrier Breakdown in Humanized Mice

**DOI:** 10.1038/srep26876

**Published:** 2016-05-31

**Authors:** Vir B. Singh, Meera V. Singh, Santhi Gorantla, Larisa Y. Poluektova, Sanjay B. Maggirwar

**Affiliations:** 1 Department of Microbiology and Immunology, University of Rochester Medical Center, 601 Elmwood Avenue, Box 672, Rochester, NY 14642, USA; 2 Department of Pharmacology and Experimental Neuroscience, University of Nebraska Medical Center, Omaha, Nebraska 68198, USA.

## Abstract

Human Immunodeficiency Virus type-1 (HIV)-associated neurocognitive disorder is characterized by recruitment of activated/infected leukocytes into the CNS via disrupted Blood Brain Barrier (BBB) that contributes to persistent neuro-inflammation. In this report, humanized NOD/scid-IL2Rγ_c_^null^ mice were used to establish that impaired Sonic hedgehog (Shh) signaling is associated with loss of BBB function and neurological damage, and that modulating Shh signaling can rescue these detrimental effects. Plasma viral load, p24 levels and CD4^+^ T cells were measured as markers of productive HIV infection. These mice also showed impaired exclusion of Evans blue dye from the brain, increased plasma levels of S100B, an astrocytic protein, and down-regulation of tight junction proteins Occludin and Claudin5, collectively indicating BBB dysfunction. Further, brain tissue from HIV^+^ mice indicated reduced synaptic density, neuronal atrophy, microglial activation, and astrocytosis. Importantly, reduced expression of Shh and Gli1 was also observed in these mice, demonstrating diminished Shh signaling. Administration of Shh mimetic, smoothened agonist (SAG) restored BBB integrity and also abated the neuropathology in infected mice. Together, our results suggest a neuroprotective role for Shh signaling in the context of HIV infection, underscoring the therapeutic potential of SAG in controlling HAND pathogenesis.

Successful control of viral replication in the combined anti-retroviral therapy (cART)-era has lead to longer and comparatively improved life for individuals infected with human immunodeficiency virus type-1 (HIV). However, persistent immune activation and inflammation puts them at a higher risk for non-AIDS related co-morbidities, including cardiovascular and HIV-associated neurological disorders (HAND). Almost 50% of HIV-infected individuals develop some form of neurological impairment in their lifetime^1^,^2^, which is clinically categorized into three types based on severity: Asymptomatic Neurocognitive Impairment (ANI), Mild Neurocognitive Disorder (MND), and HIV-Associated Dementia (HAD)^2^. The neuropathology associated with HAND is known as HIV-associated encephalitis (HIV-E), identified port-mortem by analysis of brain sections highlighting distinct neuronal loss, microglial nodules, activated resident microglia, multinucleated giant cells and infiltration predominantly by monocyte/macrophage cells^3^. Interestingly, clinical signs of neuroinflammation are more closely associated with increased numbers of activated microglia and infiltrating monocytes, rather than the viral load within the central nervous system (CNS)^4^. The Blood Brain Barrier (BBB), which is composed of closely packed brain microvascular endothelial cells, astrocyte end feet and pericytes, is critical in regulating the traffic of proinflammatory leukocytes into the CNS^5^,^6^. Studies from our lab and that of others have indicated that HIV-induced BBB dysfunction might promote the infiltration of infected/activated immune cells into CNS and contribute to excessive neuroinflammation despite reduced viral load^7^,^8^,^9^,^10^. Further, these leukocytes and their released inflammatory molecules infect/activate CNS resident microglia, macrophages and astrocytes and contribute to neurodegeneration^6^.

Lack of a suitable animal model is the biggest challenge in delineating molecular mechanisms of HAND. Current evidence of neurological complications has been obtained either from post-mortem CNS specimen derived from HIV infected individuals^11^,^12^ or from *in vivo* studies using simian immunodeficiency virus (SIV). However the major drawbacks associated with these strategies are that the post-mortem specimens provide insufficient information concerning early events leading to the neuro-cognitive impairment or the molecular mechanisms associated with HAND. The data obtained from SIV infection is not truly informative of HIV infection due to viral- strain and species-specific differences, not to mention the prohibitive costs of performing these experiments. In comparison, the humanized mouse model offers several advantages in terms of cost-effectiveness as well as recapitulation of multiple aspects of HIV infection in humans^13^. Researchers have developed a variety of humanized mouse models including NOD/scid-IL-2Rγ_c_^null^ (NSG), BALB/c-Rag2^−/−^γ_c_^−/−^, and NOD/scid BLT mice that allow successful engraftment of human CD34^+^ hematopoietic stem cells for lifetime. These mice have been used to study many aspects of HIV infection including: viremia^14^, sexual transmission and prevention^15^, ART strategies^16^, gene therapy^17^, and immune activation and dysfunction^18^. Of particular importance to our study, a report by Dash *et al.*, demonstrated that the HIV-infected, humanized mice develop neuropathology similar to that observed in HIV-infected individuals^19^, and Boska *et al.* were able to recapitulate behavioral defects in these mice, highlighting the suitability of this animal model for studying the mechanisms associated with HAND^20^.

Interestingly, an independent study by Alvarez *et al.*, established the involvement of Sonic hedgehog (Shh) signaling in maintaining the BBB integrity in wild-type mice^21^. Shh is a morphogen which, upon secretion, binds to its cognate receptor, Patched-1 (Ptc1), on the surface of target cells, thereby releasing Smoothened (Smo) to initiate downstream signaling that controls the transcription factor Gli1^22^. Alvarez *et al.* further suggested that astrocyte-derived Shh drives this signaling cascade in BBB endothelial cells and that Gli1 up-regulates the expression of tight-junction proteins and down regulates the adhesion molecules, thus, maintaining BBB integrity. These investigators showed that with respect to multiple sclerosis, Shh signaling also provides an anti-inflammatory balance to CNS-directed immune attacks. Shh is well known for its role in cell differentiation, patterning, survival, and proliferation in the developing vertebrate brain. Although, there are high levels of this molecule in the fetal brain, its expression in the adult brain is very restricted. Studies have shown that activation of the Shh signaling pathway induces proliferation and survival of neural^23^ and oligodendrocyte precursors^24^. Further, administration of recombinant Shh protein or a Shh agonist, Ag11.1, was shown to rescue spinal cord injury in adult rats^25^. Similarly, activation of Shh signaling corrected structural and cognitive deficits in a mouse model of Down syndrome when treated at birth^26^.

On the basis of these findings, we aimed to investigate the role of Shh signaling in the CNS during HIV infection by employing a humanized mouse model. Our study indicates that HIV infection results in the inhibition of Shh signaling in the CNS and thus leading to BBB damage and neuropathology. Reactivation of Shh signaling by the small molecule Smoothened Agonist (SAG), rescues HIV-induced BBB permeability via induction of Gli1 expression in endothelial cells, resulting in increased tight junction expression. Our findings implicate Shh signaling in BBB dysregulation during HIV infection, and that interventions targeting Shh signaling might prove beneficial in alleviating HIV-associated neurological impairment.

## Results

### HIV infection in humanized mice

**Figure 1:**
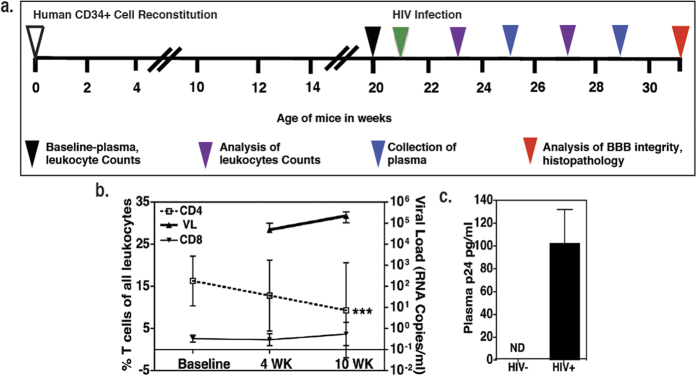
Progressive HIV infection of humanized mice. (**a**) Experimental scheme for human CD34+ cell reconstitution, HIV infection, and time frame of observations. (**b)** CD4+, CD8+ T cell percentages (N=8 per time point per group) and plasma viral load at 4 and 10 w.p.i. in HIV infected (N = 3 per time point per group). ***denotes P < 0.0001 as compared to CD4+ T cell percentages at baseline (**c)** HIV protein p24 ELISA at 10 w.p.i. (N = 4 per group).

In order to investigate how HIV infection induces BBB dysfunction we first established HIV infection in mice. As illustrated in Fig. 1a, humanized mice were infected with HIV (pNLEG1-70 GFP+, R5 tropic) at the age of 21 weeks. Productive infection was confirmed by measuring viral RNA copies in sera of mice at 4 and 10 weeks post infection (w.p.i.; N = 3 at each time point, range: 0.1–3 × 10^5^ RNA copies/ml; Fig. 1b) as well as by viral capsid protein p24 levels in plasma of mice at 10 w.p.i. (N = 4 in each group; range 73–132 pg/ml, Fig. 1c). In addition, there was a gradual depletion of CD4^+^ T cells in HIV-infected mice, which is a hallmark of HIV infection (p < 0.001 as compared to baseline, N = 8 in each group at each time point; Fig. 1b). In contrast, the numbers of CD8^+^ T cells (Fig. 1b) as well as CD19^+^ B lymphocytes (data not shown) remained comparable in infected and uninfected mice.

### HIV infection is associated with platelet activation in humanized mice

**Figure 2:**
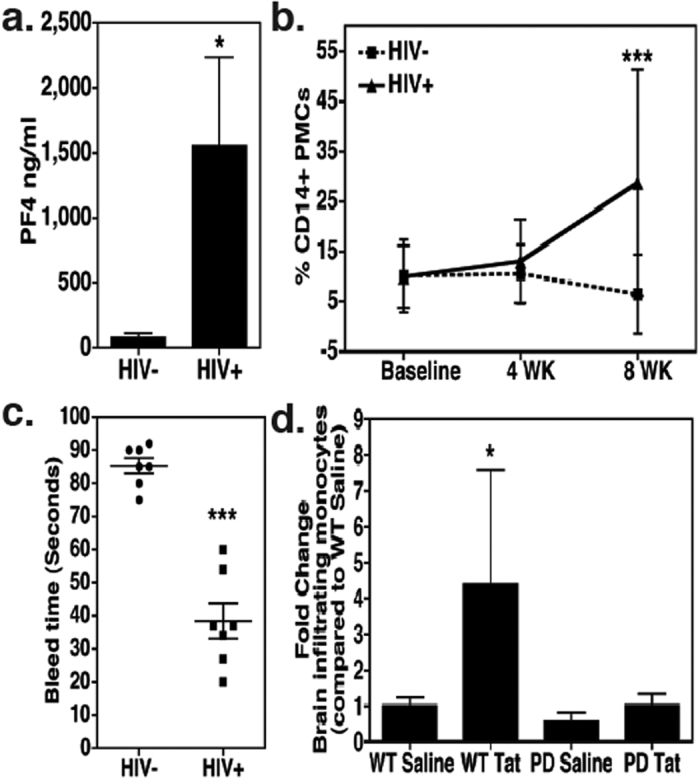
HIV infection induces platelet activation. (**a)** Plasma human PF4 levels in humanized mice measured by ELISA (N = 4 per group). (**b)** Detection of platelet-monocyte complexes (PMCs) at 4 and 8 w.p.i. in HIV-infected and uninfected humanized mice by Flow Cytometry (N = 8 per time point per group). (**c)** Tail bleed assays were performed 10 w.p.i. (N = 7 per group). (**d)** Platelet-depleted mice (N = 4) were exposed to Tat (1.5 μg/g body weight) for total 24h and migration of GFP+ monocytes (derived from CX3CR1/GFP TG mice) into the CNS was measured by flow cytometry. As shown here, Tat-induced migration of GFP+ monocytes into the CNS was completely blunted in platelet-depleted mice. *denotes P < 0.05, ***denotes P < 0.0001.

HIV-infected individuals exhibit a marked increase in platelet activation and subsequent platelet-monocyte complex (PMC) formation that leads to a pro-migratory monocyte phenotype^27^. Therefore, we sought to measure markers of platelet activation in the humanized mice by employing complementary methods. Accordingly, we measured the levels of human platelet factor 4 (PF4) by ELISA in the plasma samples derived from HIV-infected mice. PF4 is a sensitive and specific marker of platelet alpha-granule release, which is often up-regulated in patients with underlying inflammatory disorders, including those triggered by HIV-infection^28^,^29^. As shown in Fig. 2a, increased levels of circulating PF4 were noted in the infected mice (p = 0.0201,10 w.p.i. N = 4 in each group). Next, flow cytometric analyses were performed to assess the extent of PMC formation, as this reflects on the platelet activity^30^,^31^. We found that PMC numbers were increased in the blood of infected mice over time (4 to 8 w.p.i.), whereas, uninfected mice showed a declining trend of PMCs (Fig. 2b; P < 0.001 at 8 w.p.i, N = 8). Third, we performed tail bleed experiments as a functional determinant of platelet activation^7^, since several reports have indicated that chronic HIV-infection contributes to the prothrombotic abnormalities in patients, recently reviewed^32^. Consistent with this notion and results shown in Fig. 2a,b, significantly reduced clotting time was observed in infected mice at 10 w.p.i. (p < 0.0001, N = 7 in each group, Fig. 2c). Collectively, our results suggest that the HIV-infection stimulates platelet activity in humanized mice, making it possible to further study their role in HIV pathogenesis.

The pathogenic effects of HIV-infection are linked primarily to indirect actions of the viral as well as cellular factors released by infected cells^33^,^34^. Accordingly, the virally encoded protein Tat was shown to induce BBB permeability in C57BL/6 mice in a platelet-dependent manner^7^. Therefore, we speculated that the observed platelet activation in HIV-infected humanized mice might enhance the ability of monocytes to migrate into the CNS. To test this notion, we used a simplified experimental model in which normal C57BL/6 mice were exposed to platelet-depleting antibodies^35^ (which resulted into 75–80% platelet depletion – data not shown), followed by administration of highly purified Tat. The migration of syngeneically transferred monocytes (GFP+) into the CNS was then determined by flow cytometry. Significantly higher numbers of CNS-infiltrating monocytes were found upon Tat treatment as compared to saline treated mice, but only in presence of normal platelet counts (P = 0.0195, N = 4, Fig. 2d).

### HIV infection augments BBB dysfunction and neurological damage

**Figure 3:**
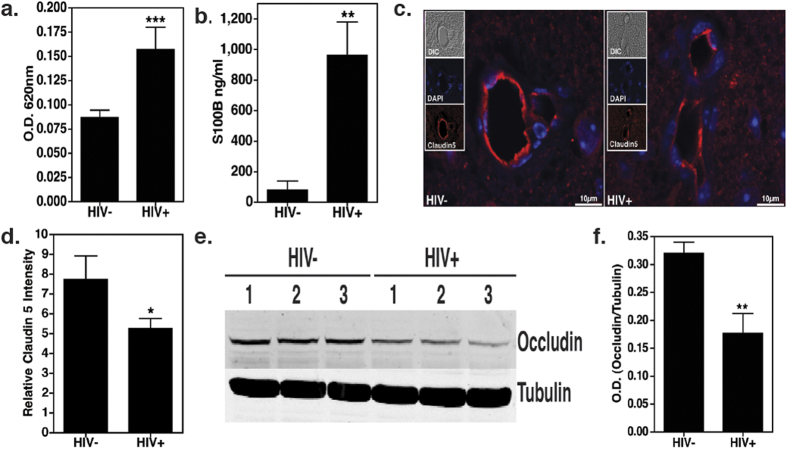
HIV infection disrupts BBB integrity. **(a**) Evans blue assay was performed to measure BBB permeability (N = 5 in each group) 10 w.p.i. Higher concentration of Evans blue dye was detected in the brains of HIV infected mice as evident by O.D. at 620 nm. (**b)** Higher levels of S100B, a CNS resident protein, were detected in peripheral blood plasma of HIV infected mice (10 w.p.i, N = 3 in each group). (**c**,**d)** Paraffin sections of brain (5 μm thick) obtained 10 w.p.i. were labeled with anti-Claudin5 (red) and counterstained with DAPI (blue) to label nuclei. Images were taken at 60X (objective magnification) and optically zoomed to 3. Scale bars are 10 μm. Relative Claudin 5 intensity was measured by dividing corrected integrated density with total area imaged from three random fields of view. These values were obtained using ImageJ software. Reduction in Claudin5 expression in vascular endothelial cells of infected mice was observed. (**e**,**f**) Immunoblot assay performed on brain lysates (N = 3 per group, 10 w.p.i.) show loss of another tight junction protein Occludin in HIV infected brains. Image J software was used to perform densitometry analysis.

To better understand how HIV induces monocyte infiltration into the CNS, we assessed if HIV infection in humanized mice leads to BBB impairment and neuropathology similar to that observed in infected individuals^11^,^12^. Here, BBB integrity was measured in humanized mice at 10 w.p.i. using Evans blue dye exclusion assay. There was a 2-fold increase in the levels of Evans blue in the brains of mice infected with HIV as compared to the uninfected control mice (P < 0.0001, N = 5, Fig. 3a). We also measured the plasma levels of S100B. This protein is mainly secreted by astrocytes and its detection in peripheral blood is an indicator of BBB disruption^36^. We detected significantly higher levels of S100B in the blood of HIV-infected humanized mice (P = 0.0012, N = 3, Fig. 3b) further suggesting a defect in BBB function.

Next, we examined the levels of two representative tight junction (TJ) proteins, Claudin5 and Occludin, that are tightly linked with the BBB function^37^,^38^. In this experiment we measured protein levels 10 w.p.i by immunohistology (IHC, p = 0.0299, Fig. 3c,d), as well as by western blot analyses (p = 0.0036, Fig. 3e,f). Both methods showed dramatic reduction in the levels of these TJ proteins in HIV-infected mice.

**Figure 4:**
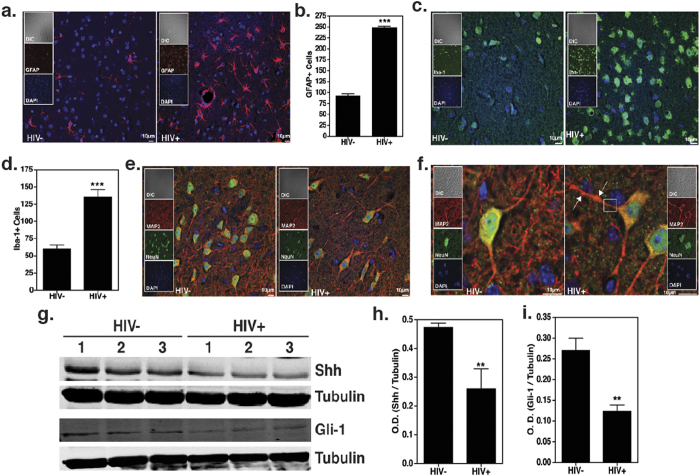
HIV infection induces neuropathology in humanized mice. Paraffin sections of brain (5 μm thick) obtained 10 w.p.i. were labeled with (**a**,**b**) anti-GFAP (red), astrocyte activation marker. Images from cortical region were taken at 40X. (**c**,**d**) anti-Iba1 (green), microglial activation marker. Images were taken at 60X. GFAP+ and Iba-1+ cells were enumerated using “particle analysis” tool from ImageJ software from three random fields of view. (**e**,**f**) Anti-MAP2 (red), anti-NeuN (green) neuronal markers. Images were taken at 60X and optically zoomed to 3 in (**f)**. **↑** indicates dendritic beading and the section enclosed in a square indicates denritic thinning. All sections were counterstained with DAPI (blue) to label nuclei. Scale bars are 10 μm. (**g**–**i**) Immunoblots showing the expression of Shh and Gli1 in the brain lysates collected from HIV infected and uninfected humanized mice (N = 3 per group). Image J software was used to perform densitometry analysis.

Additional IHC analyses of the brain tissues were conducted to measure markers of neuronal injury. HIV infected mice showed an increase in GFAP+ astrocytes (p < 0.0001, Fig. 4a,b), as well as Iba-1+ microglia (p = 0.0004, Fig. 4c,d). Additionally, MAP-2 and NeuN staining (Fig. 4e,f respectively) indicate a marked loss of synaptodendritic density and damaged dendrites are evidenced by thinning, beading and/or complete loss of dendrites.

### HIV infection is associated with down-regulation of Shh signaling in the CNS

While a lot is known about the importance of the BBB in neural homeostasis^5^,^6^, the signaling mechanisms that regulate barrier function are not well characterized. A recent report by Alvarez *et al.*^21^ was the first conclusive evidence for the role of Shh signaling in maintaining BBB function in the adult brain during multiple sclerosis. This study suggested that BBB integrity, tight junction protein expression and neuroinflammation might be directly regulated by this pathway, where in, binding of Shh to its cognate receptor Ptc1 on the surface of target cells causes Smo-mediated nuclear translocation of transcription factor Gli1, which in turn increases the expression of tight junction proteins and decreases expression of adhesion molecules^22^. Therefore, we analyzed mouse brains for expression of Shh pathway intermediates 10 w.p.i. Immunoblot analyses indicate a loss of Shh protein in HIV-infected mouse brain tissue as compared to uninfected control mice (p = 0.0065, Fig. 4g,h). Further, loss in Shh protein was correlated with loss in it’s downstream signaling molecule, Gli1 in HIV infected mice (p = 0.0017, Fig. 4g,i). Overall, these results suggest that HIV infection in humanized mice results in down-regulation of Shh signaling in the CNS.

### Restoration of Shh signaling by SAG leads to rescue of BBB function in HIV-infected, humanized mice

**Figure 5:**
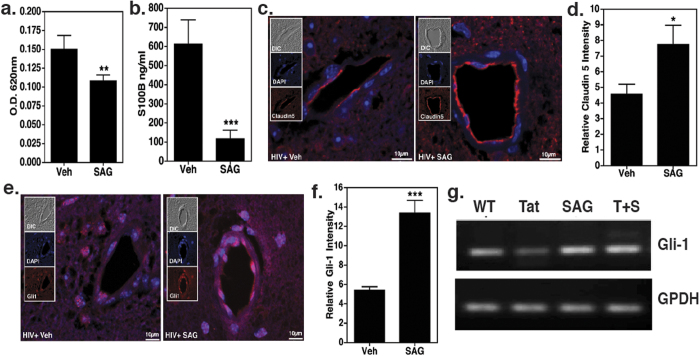
Smoothened agonist (SAG) rescues BBB integrity. (**a)** 10 w.p.i. Evans blue assay was performed on HIV infected mice either administered with SAG or vehicle (N = 3–5 per group). (**b)** Detection of S100B protein levels in plasma by ELISA (N = 3 per group). *indicates P < 0.05 and ***denotes p < 0.0001. (**c**–**f**) Paraffin sections of brain obtained 10 w.p.i. (5 μm thick) were labeled with anti-Claudin5 (red) or anti-Gli1 (red) and counterstained with DAPI (blue) to label nuclei. Images were taken at 60X for Claudin5 and 40X for Gli1 and optically zoomed to 3. Scale bars are 10 μm. Relative Claudin 5 and Gli-1 intensity was measured by dividing corrected integrated density with total area imaged from three random fields of view. These values were obtained using ImageJ software. Images show increased Claudin 5 and Gli1 expression in brain endothelial cells of SAG treated mice. (**g)** HBECs were treated with Tat (100 nM) with or without SAG (500 nM) for 24 h. RT-PCR profile of Gli1 expression indicates that Tat treatment caused a reduction in Gli1 expression and can be rescued by SAG treatment (representative gel picture from N = 3).

We asked whether fortification of BBB function via restoration of Shh signaling prevents CNS injury. To test this notion, Smo agonist, SAG, was administered to HIV infected mice. This molecule has been previously used to prevent glucocorticoid-induced neonatal cerebellar injury by normalizing Shh response^39^. In our study, 9 w.p.i., mice were injected on alternate days with SAG (20 μg/g body weight) or vehicle control, DMSO, for a total of 3 injections. At the end of the 10th week Evans blue exclusion assay was performed to measure BBB permeability. Our results showed a significant reduction in BBB permeability in mice injected with SAG (p = 0.0082, N = 3 in Vehicle control and N = 5 in SAG treated group, Fig. 5a). In addition, S100B plasma levels in SAG administered mice were significantly lower as compared to the mice that received DMSO (p = 0.0007, N = 3, Fig. 5b).

Next, we measured the levels of Gli1 and its downstream target Claudin 5 in brain endothelial cells by IHC. As shown in representative photomicrographs we observed increased staining of Claudin 5 (p = 0.0169, Fig. 5c,d) and Gli1 (p = 0.0005, Fig. 5e,f, Gli1 is autoregulated^40^) following SAG administration as compared to vehicle treated HIV infected mice. Complementary to these observations, *in vitro* treatment of Human Brain Endothelial Cells (HBECs) with Tat led to the loss of Gli1 transcripts - which was efficiently reversed by co-administration of SAG (Fig. 5g), suggesting that the effects of SAG involves restoration of Shh signaling mechanisms.

**Figure 6:**
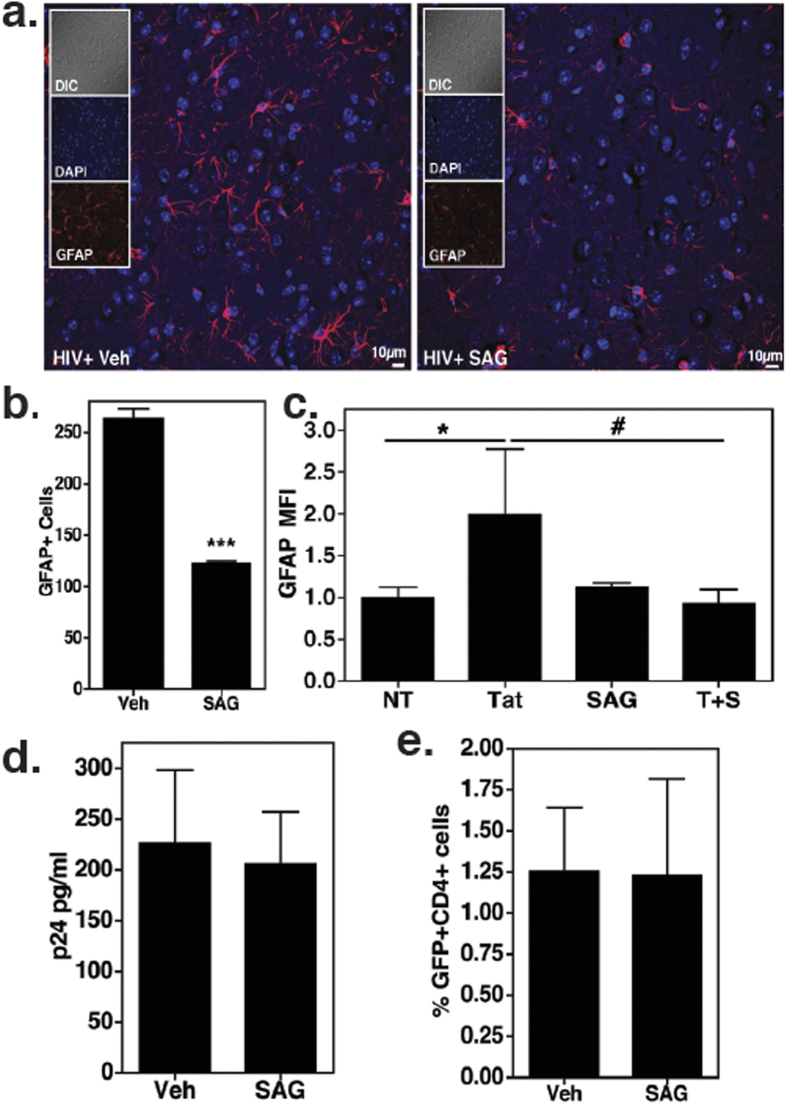
Inhibition of astrocyte activation by SAG (**a,b)**. Paraffin sections of brain obtained 10 w.p.i (5 μm thick) were labeled with anti-GFAP (red), astrocyte activation marker and counterstained with DAPI. Images were taken at 40X. Scale bars are 10 μm. GFAP+ cells were enumerated using “particle analysis” tool from ImageJ software from three random fields of view. SAG treatment caused decrease in number of GFAP expressing cells. (**c)** Primary human astrocytes were treated with Tat (100 nM) in presence or absence of SAG (500 nM, N = 4) for 24 h. GFAP expression was measured by flow cytometry. *denotes p < 0.05 as compared to untreated cells and ^#^indicates p < 0.05 as compared to Tat treated cells. (**d)** Plasma HIV p24 protein levels were detected by ELISA (10 w.p.i., N = 4 per group). (**e)** PBMCs from healthy donors were infected with HIV-GFP for 3 days and were treated with SAG or DMSO for 3 more days (N = 5). CD4+GFP+ cells were measured by flow cytometry.

Additionally, we assessed the effect of SAG on astrocyte activation, as a representative marker of CNS injury. Earlier reports have established that astrocytes respond to Shh signaling and that this pathway is important for the maintenance of communication between astrocytes and neurons^41^,^42^,^43^. Upon SAG treatment, there was a marked reduction in GFAP expressing cells in brain sections derived from HIV-infected, humanized mice (p < 0.0001, Fig. 6a,b). Further, *in vitro* treatment of primary human astrocytes with HIV Tat protein in combination with SAG showed significantly reduced GFAP expression as compared to Tat alone (Fig. 6c). Tat protein was used for *in vitro* treatment because numerous reports suggest that HIV infection induces astrogliosis via exposure to viral proteins like Tat^44^,^45^.

Since our report explores the therapeutic potential of SAG for controlling neuro-inflammation associated with HAND, complementary investigations on the effect of SAG on HIV replication were performed. As such, ELISA measurements of plasma p24 protein levels were found comparable in vehicle and SAG treated infected mice (N = 4, Fig. 6d). Secondly, primary PBMCs obtained from HIV uninfected donors were infected *in vitro* with the same GFP+ viral strain used for the mouse experiments followed by treatments with SAG or DMSO. There was no difference in percentage of CD4^+^ GFP^+^ T cells in both the treatments (N = 5, Fig. 6e), collectively indicating that SAG does not alter HIV infectivity and replication.

## Discussion

Recent clinical studies in a high risk Thai population (RV254/SEARCH 010) suggest that the invasion of CNS by HIV occurs as early as 8 days after estimated exposure, and appears to be associated with increased markers of inflammation and cellular trafficiking^46^. Upon infiltration into the CNS, it is commonly believed that these infected/activated monocytes and T-cells secrete proinflammatory cytokines such as TNF and IL-1ß, which along with viral proteins like Tat, gp120, Vpr, (collectively called HIV effector molecules) activate microglia and astrocytes. These activated cells release neurotoxic factors such as excitatory amino acids, which cause neuronal dysfunction and death that ultimately result in cognitive, behavioral, and motor deficits. Surprisingly, drug regimes with a high CNS penetration score (CPE) correlate with diagnosis of cognitive impairment in infected individuals^47^ suggesting that ART intensification would not lead to a better outcome for people with HAND.

To date, there are no effective treatment options available for HAND. Major clinical trials, including Cidofovir - a nucleoside analog effective against CMV^48^, mementin - N-methyl-D-aspartate receptor (NMDA) antagonist, widely used for treatment of neurodegenerative disorders^49^, CPI-1189, Selegiline and Minocycline - drugs with antioxidant properties^50^,^51^,^52^ while safe and well tolerated, failed to achieve significant neuroprotective benefits in persons with HIV. Disappointing outcome of these drugs clearly indicate that targeting individual factors like; oxidative stress or inflammation alone is not sufficient to control a multifaceted disorder like HAND.

Rather, prompted by an elegant report by Alvarez *et al.*, we explored the efficacy of SAG, a small molecule agonist of Shh signaling, in alleviating HIV-associated neuropathology^21^. These investigators indicated that the expression of TJ proteins on brain microvasculature endothelial cells was positively regulated by Shh signaling and that it has a prominent role in BBB differentiation and regulation of inflammatory events induced during lesion formation in multiple sclerosis. A few other studies have also showed positive benefits of SAG on rescue of spinal cord injury^25^ as well as correction of cognitive deficits in mouse model of Down syndrome^26^.

Complementary to these reports, using humanized mice as a model of HIV infection, we demonstrate that disrupted Shh signaling underlies HIV-associated BBB damage. Administration of SAG, just for a week, lead to increased expression of TJ proteins, Claudin5 and Occludin, thereby significantly improving BBB integrity. Furthermore, SAG reduced the expression of GFAP, a marker of astrocyte activation. These results are encouraging on two counts, one, improved BBB function will curb uncontrolled infiltration of infected/inflammatory leukocytes from periphery and second, reduced astrocyte activation will eventually help improve neuronal health as astrocyte dysfunction heavily contributes to HIV neuropathogenesis by affecting glutamate metabolism, secreting cytokines/chemokines and neurotoxins^53^,^54^. As mentioned previously, all of the clinically tested drugs including Cidofovir, mementin, CPI-1189 and Minocycline have failed to demonstrate improvement in cognitive function, possibly due to their limitation of targeting only one aspect of neuropathogenesis that is either oxidative stress or neuroinflammation. More specifically, none of these drugs have demonstrated any significant effect on BBB function, which if not restored, allows continuous influx of immune cells and immune factors into the CNS contributing heavily to the persistent neuro-inflammation leading to neurodegenration^5^,^6^. Thus, it is important to address multiple aspects of HAND in order to curtail persistent neuroinflammation so as to assure better clinical outcomes. Accordingly, SAG mediated induction of Shh signaling offers various advantages over above-mentioned drugs in that, in addition to dampening neuro-inflammation, it strengthens the BBB, and is also known to promote neuronal survival^24^,^25^. Thus, the observations reported herein lend a strong support to the idea that the multi-prong approach of BBB restoration, neuroprotection and neuronal survival brought about by SAG, will attenuate the neuro-inflammatory cascade associated with HIV infection and might yield beneficial outcomes in the clinical management of HAND. Additionally, whether SAG can prevent/delay the onset of neuronal damage if administered very early during HIV infection warrants further experimentation and is being actively pursued by our group.

Importantly, these findings also have significant implications for other similar complicated neurodegenerative diseases such as Alzhiemer’s Disease (AD), Parkinson’s Disease (PD) and Huntington’s disease, the progression of which is also known to be associated with neuro-inflammation and BBB impairment^55^.

## Materials and Methods

### Ethics statement

Mouse experiments were carried out in accordance with the Animal Welfare Act and the National Institute of Health (NIH) guidelines, and the University Committee on Animal Resources of the University of Rochester Medical Center approved the animal protocol (protocol #2005-161). The facilities and programs of the Vivarium and Division of Laboratory Animal Medicine of the School of Medicine and Dentistry are fully accredited by the Association for the Assessment and Accreditation of Laboratory Animal Care International (AAALAC).

The Research Subjects Review Board at the University of Rochester Medical Center approved studies involving human samples. All the study participants were adults and blood samples were obtained after written informed consent, in accordance with the Declaration of Helsinki.

### Generation of CD34-NSG mice

Humanized CD34^+^-NSG mice were generated as reported earlier^19^. Briefly, CD34^+^ cells were obtained from fetal liver and were enriched using immunomagnetic bead (CD34^+^ selection kit, #130-094-531 Miltenyi Biotech). Purity of CD34^+^ cells was measured by flow cytometry to be >90%. Further, cells were transplanted by intrahepatic injection at 10^5^ cells /mouse in 20 μl PBS using a 30 gauge needle into newly born mice that were irradiated at 1Gy using C9 cobalt 60 source (Picker corporation). The degree of engraftment and number of human cells in the peripheral blood were analyzed by flow cytometry.

### Production of infectious HIV

Virus stocks were generated by transfecting human embryonic kidney (293T) cells with pNLENG1-70 construct (R5 tropic GFP^+^ virus; a kind gift from Dr. David Levy, New York University) using polyethylenimine (PEI, #03880; Sigma-Aldrich, St. Louis, MO). Virus was concentrated using PEG6000 (#81253, Sigma-Aldrich) as described^56^, and resuspended in PBS. The 50% tissue culture infectious dose (TCID50/ml) was measured as per the ACTG Laboratory Manual Version 1.0.

### HIV infection of humanized mice

Humanized mice were infected at 20 weeks of age with HIV NLEG1-70 GFP+ virus via intraperitoneal (i.p.) injection at a single dose of 10^5^ TCID50/ml. At 4 weeks and 10 weeks post-infection (w.p.i.), levels of viral RNA copies/ml were analyzed with the automated COBAS Ampliprep system V2 (Roche Molecular Diagnostics, Mannheim, Germany) as described^19^.

### Blood Collection

Mouse peripheral blood was collected from submandibular vein using lancet (MEDIpoint) and blood was collected in EDTA microtainers (#365974, BD biosciences) at baseline and every two weeks post-infection. At terminal time point blood was collected via vena cava. Blood was either used to isolate plasma or to perform Flow cytometry to estimate cell counts for different leukocyte subtypes.

### Flow cytometry

Presence of various human cells in the peripheral blood was detected by flow cytometry as described earlier^31^. Various antibodies, including CD3 PE Cy7 (#555349), CD4 APC (#555349), CD8 PE (#555635), CD14 APC H7 (#561384), CD19 BV510 (#562953) and CD41 BV421 (#563312) were obtained from BD Biosciences (San Jose, CA). The cells were acquired using LSR II Flow Cytometer (BD Biosciences). Cells stained with only CD3, only CD14 and unstained cells were used as controls. 15000 events were acquired per sample in the leukocyte gate based on forward and side scatter. All CD14^+^ cells, which also expressed CD41, a platelet marker, were characterized as platelet monocyte complexes. The percentage of the individual cell types against total leukocyte population was numerically used to plot the graphs shown in Fig. 1.

### ELISAs

Plasma samples obtained at 10 w.p.i., and post-SAG or vehicle treatment were used to perform p24 (#5421; Advanced Biosciences Laboratories, Rockville, MD), PF4 (#DPF40, R&D Systems, Minneapolis, MN) and S100B (#708-85, Fujirebio Diagnostics, Inc, Malvern, PA) ELISAs as per manufacturer’s protocol.

### Tail Bleed assay

Tail bleed assays were performed 10 w.p.i. as described^7^. Briefly, mice were anesthetized and placed on a raised platform with tails protruding over the edge. Tails were positioned 5 mm above filter paper and a 2 mm cut was made in the tip of the tail. Time was recorded from the moment the cut was made until bleeding stopped completely.

### Isolation and quantitation of brain infiltrating leukocytes

Platelets were depleted by injecting 10–12 week old C57BL/6 mice (N = 4 per group) with a mixture of purified rat monoclonal antibodies (0.5 μg/g body weight; Emfret Analytics, Eibelstadt, Germany) that target the GPIbα receptor, found on platelets, and result in rapid Fc-independent platelet depletion in the range of 75–80% until 72 h as outlined previously^7^. After 24 h, these mice were injected with a single dose of highly purified full-length recombinant Tat (contains 1–101 amino acids; kindly provided by UR-CFAR) and incubated for next 18 h. Then, we intravenously injected these mice with highly purified GFP+ monocytes derived from CX3CR1/GFP transgenic mice. These mice constitutively express GFP in monocytic cells as well as in natural killer cells and some T cells^57^. For monocyte isolation, whole blood was obtained from vena cava and processed using EasySep Mouse Monocyte Isolation kit from StemCell Technologies as per manufacturer’s protocol. Briefly, the red blood cells were lysed using ACK RBC lysis buffer. The cells were then blocked using 5% goat serum and incubated with monocyte enrichment antibody cocktail for 15 minutes at 4 °C. The cells were then washed twice with 5% goat serum to remove unbound antibody and passed through the magnet to isolate labeled cells. The monocyte yield was 90–95% pure and all cells were GFP+. After injecting the GFP+ monocytes into the mice, we followed their migration into the CNS for 6 h. Brain infiltrating GFP+ monocytes were isolated as previously described^58^ and resuspended in 100 μl PBS. The number of infiltrating monocytes were measured based on forward and side scatter and GFP expression using Accuri C6 flow cytometer (Accuri Cytometers, Ann Arbor, MI).

### Evans blue assay

BBB integrity was determined by Evans blue exclusion assay as described 10 w.p.i^59^. Briefly, mice were injected intraperitoneally with a 2% solution of Evans blue (4 mL/kg body weight, catalog#E0197; TCI America, Portland, OR). The dye was allowed to circulate in the blood stream for 2 hours. Subsequently, mice were anaesthetized and perfused with 30 mL cold PBS through the left ventricle. After perfusion, mouse brains were collected, weighed, and homogenized in cold PBS (1:10 weight per volume). Brain homogenates were centrifuged for 25 minutes at 12,000 × g at 4 °C. Following this, an equal amount of 50% trichloroacetic acid was added to a 500 μl aliquot of brain homogenate supernatant. The samples were then incubated at 4 °C for overnight followed by centrifugation at 12,000 × g at 4 °C. The absorbance was measured at 610 nm using spectrophotometer (Spectramax M3 Multimode Microplate Reader; Molecular Devices, Sunnyvale, CA).

### Immunohistochemistry

10 w.p.i, under terminal anesthesia, mice (N = 3 in each group) were transcardially perfused with PBS followed by 4% paraformaldehyde and brains were post-fixed overnight followed by paraffin embedding. 5 μm thick sections were immuno-stained with MAP2 (catalog#AB5622; Millipore, Darmstadt, Germany; 1:500), NeuN (catalog#MAB377; Millipore, 1:250), Iba-1 (catalog#ab15690; Abcam, Cambridge, MA; 1:100), GFAP (catalog#ab7260; Abcam, 1:5000), Claudin-5 (catalog#sc28670; Santacruz, 1:100), Gli1 (sc-20687; Santacruz, 1:100) and Shh (catalog#AF464; R&D Systems, 15 μg/mL). For fluorescence labeling, species-specific AlexaFluor–tagged secondary antibodies (Alexa 488, Alexa 594, and Alexa 647; Invitrogen Life Technologies, Carrlsbad, CA) were used and nuclei were labeled with 4′, 6-diamidino-2-phenylindole (DAPI). Slides were coverslipped with ProLong Gold anti-fade reagent (Invitrogen), allowed to dry for 24 hours at room temperature in dark. Selected tissue slides were imaged in the URSMD Confocal and Conventional Microscopy Core (RRID: SciEx_12080) using an Olympus FV1000 Laser Scanning Confocal microscope (Olympus America, Center Valley, PA) with a 40X UPlan-FLN (NA1.3) or 60X (NA1.42) Plan-Apo oil objective and sequential scanning option. Selected images were optically zoomed to a maximum of 3. Confocal images were processed using Olympus FV1000 software and adjusted in the linear range to improve contrast and/or brightness. For quantification purpose, paired images were acquired on the same day using identical imaging parameters. Three-four random fields of view were captured from the cortical region per slide. Image J software was used to quantitate the confocal data. Claudin 5 and Gli-1 intensities were measured by determining integrated density in cells of interest (selected using the drawing/selection tools). The background intensity was obtained from area without any fluorescence and subtracted from the integrated density. The relative intensity of molecules of interest was calculated by dividing integrated density with the total area imaged. GFAP+ and Iba-1+ cells were enumerated using Image J “analyze particles” tool from three fields of view from respective slides.

### *In vitro* treatments

Human peripheral blood mononuclear cells (PBMCs) were isolated from HIV uninfected whole blood, resuspended in RPMI and were infected with 10^4^ TCID50/ml of pNLENG1-70 GFP^+^ virus. Three days post-infection, the cells were treated with SAG (500 nM) or DMSO as vehicle control for 3 more days. The cells were then processed for flow cytometry as described above and were stained with antibody against CD4. The percentage of CD4^+^GFP^+^ cells was compared to assess the effect of SAG (catalog#566660; EMD Millipore) on viral infection.

HBECs were cultured in a 6-well plate in DMEM medium with 10% FBS, 1% Penicillin/Streptomycin, and 1 μg/mL hydrocortisone. Cells were treated with HIV Tat (100 nM) with or without SAG (500 nM) for 24 hours. Cells were either processed for protein extraction or RNA extraction. For protein, cells were lysed in protein lysis buffer (50 mM HEPES (pH 7), 250 mM NaCl, 0.1% Nonidet P-40, 5 mM EDTA, 10 mM NaF, 0.1 mM Na_3_VO_4_, 50 μM ZnCl_2_, supplemented with 0.1 mM PMSF, 1 mM DTT, and a mixture of protease and phosphatase inhibitors) and incubated on ice for 15 min. For RNA, cells were lysed in Trizol (catalaog #15596-026; Invitrogen) and RNA extraction was performed as per the manufacturer’s protocol.

Human primary astrocytes (kindly provided by Dr. Anuja Ghorpade, University of North Texas, USA) were cultured in a 6-well plate in DMEM/F12 medium with 10% FBS, 1% Penicillin/Streptomycin. Cells were treated with HIV Tat (100 nM) with or without SAG for 24 hours. Protein was extracted from these cells using protein lysis buffer, as mentioned earlier.

### Reverse Transcriptase –Polymerase Chain Reaction (RT-PCR) assay

Total cellular RNA from differently treated HBECs was subjected to DNase I (catalog #18068-015; Invitrogen) treatment for 15 min at room temperature followed by DNase I inactivation by adding 1 μl of 24 mM EDTA solution and incubating the mixture for 10 min at 65 °C. DNase treated RNA (1 μg) was used for cDNA synthesis using iScript cDNA Synthesis Kit (catalog#170-8890; Bio-Rad, Hercules, CA) as per the manufacturer’s protocol. For end-point PCRs 1 μl of cDNA were amplified using 2U of Taq DNA polymerase (catalog #10342-020; Invitrogen), 0.2 mM dNTPs, 1.5 mM MgCl_2_ and 500 nM forward and reverse primers. The primers used in the experiments were, Gli1VSF1- GAAGGAGTTCGTGTGCCACT and Gli1VSR1- AGGTTTTCGAGGCGTGAGTA.

### Western blot analysis

Mouse brains from 10 w.p.i were used to make lysates (N = 3 per group). Cell lysates (20–30 μg) were separated on 7.5–12% polyacrylamide gels under denaturing conditions. Proteins were electrophoretically transferred onto nitrocellulose membrane (catalog #88018; Thermo Scientific) and blocked for 1 h, followed by incubation with primary antibodies raised against Gli1 (rabbit polyclonal; catalog #SC-20687; Santa Cruz), Sonic hedgehog (rabbit monoclonal; catalog#2207; Cell Signaling Technology, Danvers, MA), Occludin (goat polyclonal; catalog #sc-8145; Santa Cruz) and α-Tubulin (mouse monoclonal; catalog#sc-8035; Santa Cruz). Species-specific IRDye-conjugated secondary antibodies (1:20,000, LI-COR BioSciences, Lincoln, NE) were used to detect the specific antigen-antibody interactions on the membrane. Membranes were imaged using Odyssey infrared imaging system (LI-COR BioSciences). Image J software was used for densitometry analysis and normalized to the optical density of the loading control, Tubulin.

### Statistical analysis

Graphpad Prism version 4 was used to perform all statistical analyses. The comparison between different whole blood cell percentages in HIV infected and uninfected mice at different time points was performed using 2-way ANOVA. Comparisons between BILs in WT and platelet depleted mice with and without Tat were done by 2-way ANOVA. All other comparisons were done using unpaired t test. Statistical significance is indicated in the figures as *p < 0.05, **p < 0.01, and ***p < 0.001.

## Additional Information

**How to cite this article**: Singh, V. B. *et al.* Smoothened Agonist Reduces Human Immunodeficiency Virus Type-1-Induced Blood-Brain Barrier Breakdown in Humanized Mice. *Sci. Rep.* **6**, 26876; doi: 10.1038/srep26876 (2016).
